# Bone Metastases of Breast Cancer in the Skull Base: An Unusual Metastatic Spread

**DOI:** 10.7759/cureus.97171

**Published:** 2025-11-18

**Authors:** Mariana S Beja, Joana Marques, Vasco S Cardoso, Zacharoula Sidiropoulou

**Affiliations:** 1 Obstetrics and Gynecology, Unidade Local de Saúde (ULS) - Lisboa Ocidental, Lisbon, PRT; 2 General Surgery, Unidade Local de Saúde (ULS) - Lisboa Ocidental, Lisbon, PRT; 3 General Surgery, Unidade Local de Saúde Almada - Seixal, Lisbon, PRT

**Keywords:** advanced-stage breast cancer, breast cancer, elderly patient management, skull base metastases, solitary bone metastasis

## Abstract

Breast cancer is a prevalent malignancy and a leading cause of cancer-related mortality among women. A subset of women present with primary disseminated disease, with bone being a frequent site of metastatic involvement. However, metastatic spread to the base of the skull is rare, asymptomatic, and often difficult to diagnose. The authors present a case of an elderly female patient in her early 80s with advanced breast cancer who had bone metastases limited to the base of the skull at the time of diagnosis.

## Introduction

Breast cancer is the most frequently diagnosed cancer among women, accounting for 12% of all cancers [1]. Despite significant improvements in early detection through screening programs and advances in treatment, it remains the leading cause of cancer-related death in women worldwide [1]. Approximately 6-10% of women are diagnosed with primary disseminated breast cancer, which has a five-year survival rate of only 27% [2,3]. The most common sites of breast cancer metastasis are the bones (60%-75%), lungs (32%-37%), liver (32%-35%), and brain (up to 10%) [4]. Bone-only metastases, with no evidence of involvement in other organs, occur in 25%-40% of women with metastatic disease, often affecting multiple skeletal sites [5]. However, solitary bone metastases, confined to a single skeletal site, are rare and typically involve the spine, pelvic bones, or sternum [5,6]. Skull base metastases are often asymptomatic and are usually detected only after the development of cerebral neuropathy or craniofacial pain [7,8]. Still, different clinical syndromes can occur depending on the anatomical location of the lesion [7].

## Case presentation

A previously autonomous female patient in her early 80s, with a good performance status, was admitted to our tertiary hospital for an etiological investigation of polyarthritis. Her medical history included atrial fibrillation, congestive heart failure, hypertension, chronic kidney disease, and osteoporosis. The patient was widowed and currently lived with her son.

The patient exhibited a six-month history of inflammatory symmetrical polyarthritis, predominantly affecting small joints. Despite treatment with low-dose prednisolone, her symptoms had progressively worsened. Although the clinical and laboratory findings were consistent with rheumatoid arthritis, a thoraco-abdominal-pelvic computed tomography (CT) scan was performed to exclude paraneoplastic polyarthritis, due to the older age of onset and lack of response to treatment. The CT scan revealed a suspicious lesion in the right breast.

The patient reported a palpable and painless breast mass that had been present for several years. However, no specific imaging was performed during this period, nor had she undergone routine breast cancer screening.

The patient's obstetric and gynecological history included three pregnancies, one of which resulted in neonatal death. Menarche occurred at the age of 13, and menopause at the age of 50. The patient's family history was otherwise unremarkable.

Following the identification of a suspicious breast lesion on the CT scan, a thorough clinical examination was performed in our department. This revealed a palpable lesion in the upper outer quadrant of the right breast, without any skin changes. No enlarged axillary or supraclavicular lymph nodes were detected, and the contralateral breast showed no additional lesions.

Given the clinical suspicion, bilateral mammography and breast ultrasound were performed, revealing a dense, irregular, spiculated, hypoechogenic lesion in the upper outer quadrant of the right breast, measuring approximately 36 mm. No other suspicious lesions were identified in either breast or any axillary lymph nodes. Subsequently, an ultrasound-guided biopsy of the lesion was performed, and the histopathological examination confirmed the presence of invasive breast carcinoma, no special type (NST). Immunohistochemical analysis revealed the expression of hormone receptors (100% estrogen receptor and 60% progesterone receptor), negative human epidermal growth factor receptor 2 (HER-2), and proliferation marker Ki-67 in less than 10% of the cells.

Laboratory results revealed normocytic anemia and an elevated alkaline phosphatase level. Additionally, the patient's tumor markers, including carcinoembryonic antigen (CEA) and cancer antigen (CA) 15.3, were within normal limits. The patient’s laboratory results are summarized in Table 1.

**Table 1:** Relevant laboratory findings Laboratory investigations revealed elevated rheumatoid factor and anti-cyclic citrullinated peptide (anti-CCP) antibodies, consistent with rheumatoid arthritis. Alkaline phosphatase was also elevated, possibly reflecting increased osteoblastic activity, which can be associated with bone metastasis. Tumor markers CA15.3 and CEA, commonly used in the diagnosis and monitoring of breast cancer, were within normal limits; yet these may remain negative even in the presence of metastatic disease.

Parameter	Value	Reference Range
Hemoglobin	9.9 g/dL	12.0-15.0 g/dL
Mean Corpuscular Volume	83.8 fL	80.0-96.1 fL
Iron	40 µg/dL	33-193 µg/dL
Ferritin	251 ng/mL	30-340 ng/mL
Alkaline Phosphatase	132 U/L	35-104 U/L
Rheumatoid Factor (RF)	27 IU/mL	<15 IU/mL
Antinuclear Antibody (ANA)	Negative	
Anti–Cyclic Citrullinated Peptide (anti-CCP)	962 U/mL	<5 U/mL
Human leukocyte antigen B27 (HLA-B27)	Negative	
Extractable Nuclear Antigen (ENA)	Negative	
Carcinoembryonic Antigen (CEA)	<1,80 ng/mL	<5 ng/mL
Cancer Antigen 15.3 (CA 15.3)	19.3 U/mL	<31.3 U/mL

**Figure 1:**
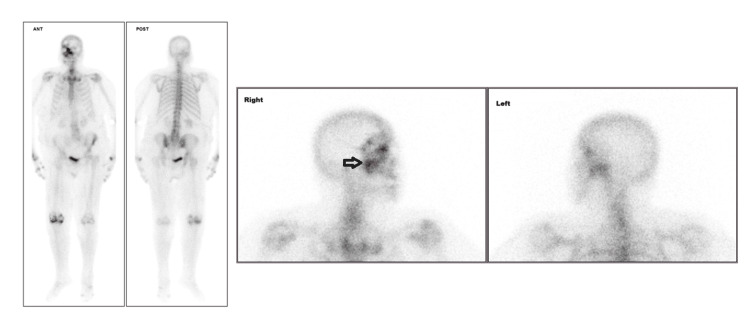
Bone metastases in the right hemiface in bone scintigraphy Bone scintigraphy images reveal an intense focus of increased radiotracer uptake in the right hemiface, displaying a pattern that strongly suggests bone metastasis. There is also heterogeneous radiotracer activity distribution along the dorsal and lumbar spine, as well as mildly increased uptake at the shoulders, sternoclavicular joints, hands, and knees (more pronounced on the right), findings more consistent with degenerative or inflammatory osteoarticular pathology. The remaining skeletal distribution of the radiopharmaceutical is within the expected range for the patient’s age.

The thoraco-abdominal-pelvic CT scan, initially performed to exclude paraneoplastic polyarthritis, revealed a large spiculated mass in the right breast. However, the CT scan did not reveal any suspicious adenopathy or distant metastases. Despite the absence of clinically positive axillary lymph nodes, a large tumor, or an aggressive tumor biology, the progressive worsening of the patient’s polyarthritis raised the concern for the presence of bone metastases. Consequently, additional studies were conducted to assess for distant metastases. Bone scintigraphy revealed a highly suggestive pattern of bone metastases in the right hemiface (Figure 1).

However, the patient exhibited no symptoms, and no palpable mass or cranial nerve palsy was identified. To further evaluate the suspected bone metastases, a CT scan of the skull and face was performed, revealing erosive lesions at the base of the skull involving the sphenoid and occipital bones.

Based on these findings, the patient's breast cancer was staged as cT2N0M1, stage IV. After discussion with the multidisciplinary team, palliative therapy with fulvestrant and ribociclib was initiated.

Eighteen days later, following a prolonged hospitalization for acute pyelonephritis, the patient developed severe mobility impairment and became bedridden at home. Consequently, her treatment plan was revised: fulvestrant and ribociclib were discontinued, and she started endocrine therapy with the aromatase inhibitor, letrozole.

One month later, the patient's condition continued to deteriorate, and she was readmitted to the hospital due to prostration and refusal to eat. Due to altered consciousness, a cranioencephalic CT scan was performed, revealing new bilateral frontal expansile cortico-subcortical lesions suggestive of brain metastases.

Considering the patient's age, comorbidities, and advanced stage of illness, palliative radiotherapy for intracranial metastases was proposed, but the patient died before treatment could be initiated.

## Discussion

This report describes a case of a patient with advanced breast cancer who presented with bone-only metastases at the skull base. This unusual metastatic site, combined with the absence of other metastatic lesions, made the diagnosis particularly challenging.

Although the patient was diagnosed with luminal A breast cancer, which accounts for approximately 60%-70% of all breast cancer cases and is typically associated with a favorable prognosis, she presented advanced clinical disease, which has a five-year survival rate of only 27% [2,3,9].

The presence of bone metastases has been demonstrated to have a significant impact on both overall survival and health-related quality of life, due to the associated symptoms such as pain, fatigue, and skeletal-related events [10]. Bone metastases are more prevalent in low-grade, estrogen receptor-positive tumors and in tumors larger than 2 cm. Additional risk factors include lymph node involvement at presentation and younger age, neither of which was observed in this case [11].

Despite the development of diagnostic imaging techniques that can detect asymptomatic solitary metastases, bone metastases that are confined to a single skeletal site remain uncommon. Parkes et al. analyzed 1,445 patients with metastatic breast cancer with bone-only metastases, of whom 290 patients (20%) had a single bone metastasis. The spine was the most frequently affected site, with only 1% of patients having a metastasis in the skull [5].

Skull base metastases from distant tumors occur in approximately 4% of cancer patients, with breast cancer being the most prevalent cause among women. However, autopsy series suggest that this condition may be an underestimate. These metastases typically manifest as a late event in the course of the disease, often following the development of other bone metastases [7].

Greenberg et al. identified five clinical syndromes associated with skull base metastases, according to their anatomical location: orbital, parasellar, middle fossa, jugular foramen, and occipital condyle [12]. Nevertheless, skull base metastases frequently remain asymptomatic until their growth causes pain or cranial nerve palsy [7]. Cranial neuropathy develops in 21% of patients and typically indicates a poorer prognosis [7,13].

Bone scintigraphy is the most commonly used conventional imaging technique for detecting bone metastases, showing metabolically active metastases. Despite its high sensitivity, bone scintigraphy has lower specificity and higher false-positive rates [14]. In our case, the presence of a single, intense focus on the right hemiface made it essential to perform additional imaging studies, such as a CT scan, to confirm the presence of skull base metastases. For skull base metastases, a CT scan of the skull with bone windows is the best method to visualize bone lesions, which are generally lytic in appearance [7]. Positron emission tomography integrated with CT (PET/CT) has been shown to have a significantly higher lesion-based sensitivity and is also useful for detecting early bone metastases [15,16]. Magnetic resonance imaging (MRI) may also be required to demonstrate dural involvement, as well as intracranial and subcranial extension [13]. Although PET/CT was initially considered and offers superior diagnostic accuracy, the sequential bone scintigraphy followed by a confirmatory CT approach successfully achieved accurate diagnosis and appropriate treatment planning in this case.

Occasionally, a biopsy may be required to confirm the diagnosis [13]. However, given the patient's life expectancy and the morbidity associated with invasive procedures, a transsphenoidal biopsy or surgical approach was deemed inappropriate in this case.

Regarding the treatment of skull base metastases, surgery is reserved for a select group of patients [7,13]. For symptomatic lesions, radiotherapy is considered the standard treatment, as it can effectively reduce craniofacial pain and, in many cases, lead to regression of cranial nerve paralysis [7].

The diagnosis and management of breast cancer in elderly patients present unique challenges that require a personalized approach. Although elderly patients may experience similar benefits from treatment as younger patients, they may be at an increased risk for severe treatment-related toxicities; therefore, careful patient selection and rigorous monitoring are essential in this population. Given our patient's multiple comorbidities but preserved performance status and functional independence, and following comprehensive clinical assessment principles recommended for elderly cancer patients, first-line palliative treatment with a CDK4/6 inhibitor in combination with endocrine therapy was initiated, as they are safe and effective drugs for the elderly population [17-19].

In our case, primary endocrine resistance was observed, with disease progression occurring within the first six months of first-line therapy. The selection of second-line treatment - whether further endocrine-based therapy or chemotherapy - should be guided by disease aggressiveness, extent of metastasis, and organ function. It should also consider the toxicity profiles of the available options [20].

## Conclusions

This case highlights the diagnostic challenges posed by skull base metastasis in breast cancer, especially when the patient is asymptomatic upon presentation. The presence of craniofacial pain or unilateral cranial nerve palsy should prompt suspicion of bone metastases in this region. Although biopsy remains the gold standard for confirming metastatic lesions, the potential morbidity associated with invasive procedures in this anatomically complex area, especially for elderly patients, must be carefully considered. This case reinforces the importance of a personalized and multidisciplinary approach in breast cancer patients, particularly when dealing with atypical metastatic presentations.
